# Aortic stiffness effectively risk stratifies diabetic patients with suspected myocardial ischemia undergoing vasodilatory stress perfusion cardiac magnetic resonance

**DOI:** 10.1186/s12872-023-03532-0

**Published:** 2023-10-10

**Authors:** Sukanda Pengyos, Thananya Boonyasirinant, Yodying Kaolawanich

**Affiliations:** 1 Division of Cardiology, Department of Medicine, Faculty of Medicine Siriraj Hospital, Mahidol University, Bangkok, 10700 Thailand; 2 Division of Cardiology, Department of Medicine, Rajavithi Hospital, Bangkok, Thailand

**Keywords:** Aortic stiffness, Cardiovascular magnetic resonance, Coronary artery disease, Diabetes mellitus, Major adverse cardiovascular events, Pulse wave velocity, Prognostic value

## Abstract

**Background and aims:**

Cardiovascular magnetic resonance (CMR) comprehensively assesses aortic stiffness and myocardial ischemia in a single examination. Aortic stiffness represents a subclinical marker of cardiovascular risk in the general population, including patients with diabetes mellitus. However, there is no prognostic data regarding aortic stiffness in patients with diabetes mellitus undergoing stress perfusion CMR.

**Methods:**

Consecutive patients with diabetes mellitus with suspected myocardial ischemia referred for adenosine stress perfusion CMR with aortic pulse wave velocity (PWV) during 2010–2013 were studied. The primary outcome was major adverse cardiovascular events (MACE), defined as the composite of cardiac mortality, nonfatal myocardial infarction (MI), hospitalization for heart failure, coronary revascularization (> 90 days post-CMR), and ischemic stroke. The secondary outcome was hard cardiac events, defined as the composite of cardiac mortality and nonfatal MI.

**Results:**

A total of 424 patients (median follow-up 7.2 years) were included. The mean PWV was 12.16 ± 6.28 m/s. MACE and hard cardiac events occurred in 26.8% and 9.4% of patients, respectively. Patients with elevated PWV (> 12.16 m/s) had a significantly higher incidence of MACE (HR 2.14 [95%CI 1.48, 3.09], p < 0.001) and hard cardiac events (HR 2.69 [95%CI 1.42, 5.10], p = 0.002) compared to those with non-elevated PWV. Multivariable analysis demonstrated that PWV independently predicts MACE (p = 0.003) and hard cardiac events (p = 0.01). Addition of PWV provided incremental prognostic value beyond clinical data, left ventricular mass index, myocardial ischemia, and late gadolinium enhancement in predicting MACE (incremental χ² 7.54, p = 0.006) and hard cardiac events (incremental χ² 5.99, p = 0.01).

**Conclusions:**

Aortic stiffness measured by CMR independently predicts MACE and hard cardiac events and confers significant incremental prognostic value in patients with diabetes mellitus with suspected myocardial ischemia. Aortic stiffness measurement could potentially be considered as part of a stress perfusion CMR protocol to enhance risk prediction in patients with diabetes mellitus.

**Supplementary Information:**

The online version contains supplementary material available at 10.1186/s12872-023-03532-0.

## Introduction

Patients with diabetes mellitus are at an increased risk of developing coronary artery disease (CAD) and experiencing future cardiovascular events. Despite significant advances in diabetes management and care, diabetes remains associated with considerable mortality, with the primary cause of cardiovascular death being CAD [1]. Accordingly, early diagnosis and risk stratification of CAD in patients with diabetes mellitus are essential for improving patient outcomes. Cardiovascular magnetic resonance (CMR) imaging is a reliable and comprehensive diagnostic modality that can be used to assess patients with known or suspected CAD. CMR provides important data specific to ventricular function, stress perfusion, and late gadolinium enhancement (LGE), and these imaging parameters had been demonstrated to have strong prognostic value [2].

Aortic stiffness is a subclinical marker of cardiovascular risk in both general population and in patients with diabetes mellitus [3, 4]. Measurement of the carotid-femoral pulse wave velocity (PWV) is considered the gold standard for evaluating aortic stiffness [5, 6]. However, CMR is often the preferred method. CMR-based aortic PWV measurements have been well validated (compared with invasive pressure recordings) with high reproducibility [7]. An important benefit of CMR is its ability to provide cross-sectional images of the entire length of the aorta with high spatial resolution, and aortic length measurement is performed directly without the need for geometric distance assumptions [8].

Furthermore, and importantly, CMR can assess PWV and perform a stress perfusion test in a single examination. We recently demonstrated the association between aortic stiffness and myocardial ischemia, as well as the prognostic value of aortic stiffness using CMR [9, 10]. Swoboda et al. demonstrated that CMR-based PWV was associated with poor glycemic control and adverse cardiovascular events in asymptomatic patients with type 2 diabetes mellitus [11]. However, no specific data exists regarding the value of aortic stiffness in predicting cardiovascular events for patients with diabetes mellitus undergoing vasodilatory stress perfusion CMR. Therefore, this study aims to investigate the prognostic value of aortic stiffness using CMR-based PWV in patients with diabetes mellitus presenting with suspected myocardial ischemia.

## Methods

### Study population

We retrospectively enrolled consecutive patients with diabetes mellitus referred for adenosine stress perfusion CMR with PWV to evaluate suspected myocardial ischemia. The study was conducted at the Division of Cardiology, Department of Medicine, Faculty of Medicine Siriraj Hospital, Mahidol University, Bangkok, Thailand, from 2010 to 2013. At our institution, the assessment of aortic stiffness using PWV is a standard component of our comprehensive CMR protocol for CAD evaluation. All patients were aged > 18 years, and the diagnosis of diabetes mellitus was established according to the current American Diabetes Association guidelines [12]. Patients with one or more of the following conditions were excluded from the study: (1) patients with aortic diseases that could potentially influence PWV measurements, such as thoracic aortic aneurysm or aortoiliac occlusive disease; (2) incomplete CMR exams; (3) patients with missing or incomplete follow-up data; and/or (4) the presence of any serious concomitant disease expected to limit life expectancy.

Detailed medical history and medications were collected on the day of the CMR study. Diagnosis of hypertension, hyperlipidemia, CAD, and ischemic stroke was defined according to recent guidelines [13–16]. Electrocardiography (ECG) and laboratory results, including fasting plasma glucose and hemoglobin A1c (HbA1c), were obtained from the medical records within 3 months before CMR.

This study was approved by Siriraj Institutional Review Board (SIRB) (COA no. Si 782/2016), Faculty of Medicine Siriraj Hospital, Mahidol University. The need for consent was waived by the board due to its retrospective nature and as all personal identifying information was obliterated. The study protocol conforms to the ethical guidelines of the 1975 Declaration of Helsinki.

### CMR protocol

CMR was performed to assess cardiac function, stress perfusion, LGE, and PWV using a 1.5 T Philips Achieva XR Scanner (Philips Medical Systems, Best, the Netherlands). A cardiac functional study was performed using images acquired via a standard retrospective ECG-gated cine balanced steady-state free precession (SSFP) sequence in multiple short- and long-axis views. The image parameters were spatial resolution 1.5 × 1.5 × 8.0 mm, 10–12 slices, gap 0 mm, sensitivity encoding factor 2, repetition times (TR) 3.3 and 2.7 ms, echo times (TE) 1.6 and 1.3 ms, field of view (FOV) 270 × 320 mm, and flip angle 60 degrees.

The myocardial first-pass perfusion study was performed by injection of 0.05 mmol/kg of gadolinium contrast agent (Magnevist; Bayer Schering Pharma, Berlin, Germany) at 4 ml/s immediately after a 4-minute infusion of 140 mcg/kg/min of adenosine. At least three short-axis slices of basal, mid, and apical left ventricular (LV) levels were acquired using an ECG-triggered, SSFP, inversion-recovery, single-shot, turbo gradient-echo sequence. The image parameters were TR 2.6 ms, TE 1.32 ms, FOV 270 × 320 mm, and flip angle 50 degrees.

PWV images were acquired during the waiting period between the stress and LGE imaging and determined with the free-breathing, velocity-encoded CMR (VE-CMR) technique as through-plane flow in the mid-ascending and mid-descending thoracic aorta at the level of the pulmonary trunk. The imaging parameters were retrospective ECG trigger, TR 5.3 ms, TE 3.1 ms, flip angle 12 degrees, FOV 250 × 210 mm, slide thickness 8 mm, matrix 2.0 × 2.0 mm, reconstructed spatial resolution 1.12 × 1.12 mm, temporal resolution 10–20 ms, and velocity encoding 170 cm/s [17].

LGE images were acquired approximately 10 min after administration of an additional bolus of gadolinium (0.1 mmol/kg, rate 4 ml/s) via a 3D segmented gradient-echo inversion-recovery sequence. The images were acquired in multiple short- and long-axis views similar to the functional images. The parameters for the LGE study were TR 4.1 ms, TE 1.25 ms, flip angle 15 degrees, FOV 303 × 384 mm, matrix 240 × 256, in-plane resolution 1.26 × 1.5 mm, and 1.5 sensitivity-encoding factor.

### Image analysis

Standard LV volumes, mass, and ejection fraction (EF) were quantitatively measured from the stack of short-axis SSFP cine images [18]. The perfusion and LGE images were analyzed using visual assessment and consensus by two CMR-trained physicians blinded to clinical and follow-up data. Segmentation of each slice was performed following the recommendations of the American Heart Association [19]. Perfusion images were read, and each of the 16 segments was visualized (segment-17 at the apex was not visualized). Myocardial ischemia was defined as a subendocardial perfusion defect that could potentially extend to the subepicardium and met the following criteria [1] persisted beyond peak myocardial enhancement and for several RR intervals, [2] was more than two pixels wide, [3] followed one or more coronary arteries, and [4] showed absence of LGE in the same segment [20]. LGE images, including subendocardial or transmural LGE, were also subjected to visual assessment [21]. LGE was considered present only if confirmed on both the short-axis and at least one other long-axis view [21].

### PWV analysis [17]

The PWV analysis was performed using EasyVision 5.2 (Philips Medical Systems, Best, the Netherlands), separate from the functional, perfusion, and LGE studies. (Supplemental Fig. 1). Contours of mid-ascending and mid-descending thoracic aorta were drawn manually to achieve the flow (m/s) at both locations throughout all phases of the cardiac cycle. The corresponding flow-time curve was generated. Pulse wave arrival time was measured as the intersection point of the linear extrapolation of the baseline and the steep early systolic stage, while aortic path length was determined by multiplanar reconstruction of axial half-Fourier acquisition from the steady-stage image. The reconstructed sagittal view of the path length was depicted as the centerline from the levels of the mid-ascending to the mid-descending thoracic aorta, and corresponding to the same level obtained on VE-CMR.

The PWV between the mid-ascending and mid-descending thoracic aorta was calculated using the following formula: PWV = Δ x / Δ T (ms).

Where Δ x reflects the length of the aortic path between the mid-ascending and mid-descending thoracic aorta, and Δ T represents the time delay between the arrival of the foot of the pulse wave at these two corresponding levels. Our research group reported excellent intraobserver and interobserver agreement for PWV measurement [17].

### Clinical follow-up

Follow-up data were collected from clinical visits, medical records, or contact with the patient’s physician. Event adjudication was blinded to clinical and CMR data. The prespecified primary outcome was major adverse cardiovascular events (MACE), which was defined as the composite of cardiac mortality, nonfatal myocardial infarction (MI), hospitalized for heart failure, coronary revascularization, and ischemic stroke. The secondary outcome was hard cardiac events, which was defined as the composite of cardiac mortality and nonfatal MI. Cardiac mortality was defined as all deaths in the setting of CAD, congestive heart failure, and sudden cardiac death [22]. MI was defined in accordance with the joint European Society of Cardiology/American College of Cardiology consensus document for the definition of MI [23]. Need for coronary revascularization within 90 days after CMR was considered to have been triggered by the results of CMR, so they were censored from the analysis.

### Statistical analysis

Statistical analysis was performed using SPSS Statistics version 22.0 (SPSS Inc., Chicago, IL, USA). Continuous data are presented as mean ± SD or median and interquartile range (IQR), as appropriate. Categorical variables were presented as absolute numbers and percentages. Continuous data were compared using the two sample t-test or the Mann–Whitney U test. Categorical data were compared using χ2 tests or Fisher’s exact tests as appropriate. Elevated and non-elevated PWV were defined as values above and below the mean of the entire cohort, respectively. To analyze the predictors of elevated PWV, we conducted a binary logistic regression analysis to evaluate univariable predictors based on baseline characteristics and CMR parameters. Variables with a p-value < 0.05 in the univariable analysis were subsequently included in the multivariable analysis. The Kaplan-Meier method was utilized to estimate composite outcomes for MACE and hard cardiac events in both patients with elevated and non-elevated PWV, as well as among PWV tertiles. These estimates were then compared using the log-rank test. To analyze the predictors of MACE and hard cardiac events, a Cox regression analysis was performed to assess univariable predictors from baseline characteristics and CMR parameters. Variables with a p-value < 0.05 in the univariable analysis were subsequently included in the multivariable analysis. To evaluate the incremental prognostic values of predictors for MACE and hard cardiac events, global chi-square values were calculated after adding predictors in the following order: clinical variables only, and then clinical + CMR variables. The clinical and CMR variables were derived from factors previously identified as independent predictors for MACE and hard cardiac events. Patients were subsequently divided into four groups based on the presence or absence of myocardial ischemia and elevated or non-elevated PWV. Cox regression analyses were employed to assess the relationship between the four patient groups: negative ischemia-non-elevated PWV, positive ischemia-non-elevated PWV, negative ischemia-elevated PWV, and positive ischemia-elevated PWV, and their associated outcomes. The Bonferroni-Holm method was applied to account for multiple pairwise comparisons. Finally, Cox regression analysis for MACE was performed in patients with elevated and non-elevated PWV across ten prespecified subgroups: female and male, above and below the mean age, above and below the mean body mass index (BMI), above and below the mean HbA1c, and with and without known CAD. All statistical tests were two tailed and p < 0.05 was regarded as significant.

## Results

### Study population

**Fig. 1:**
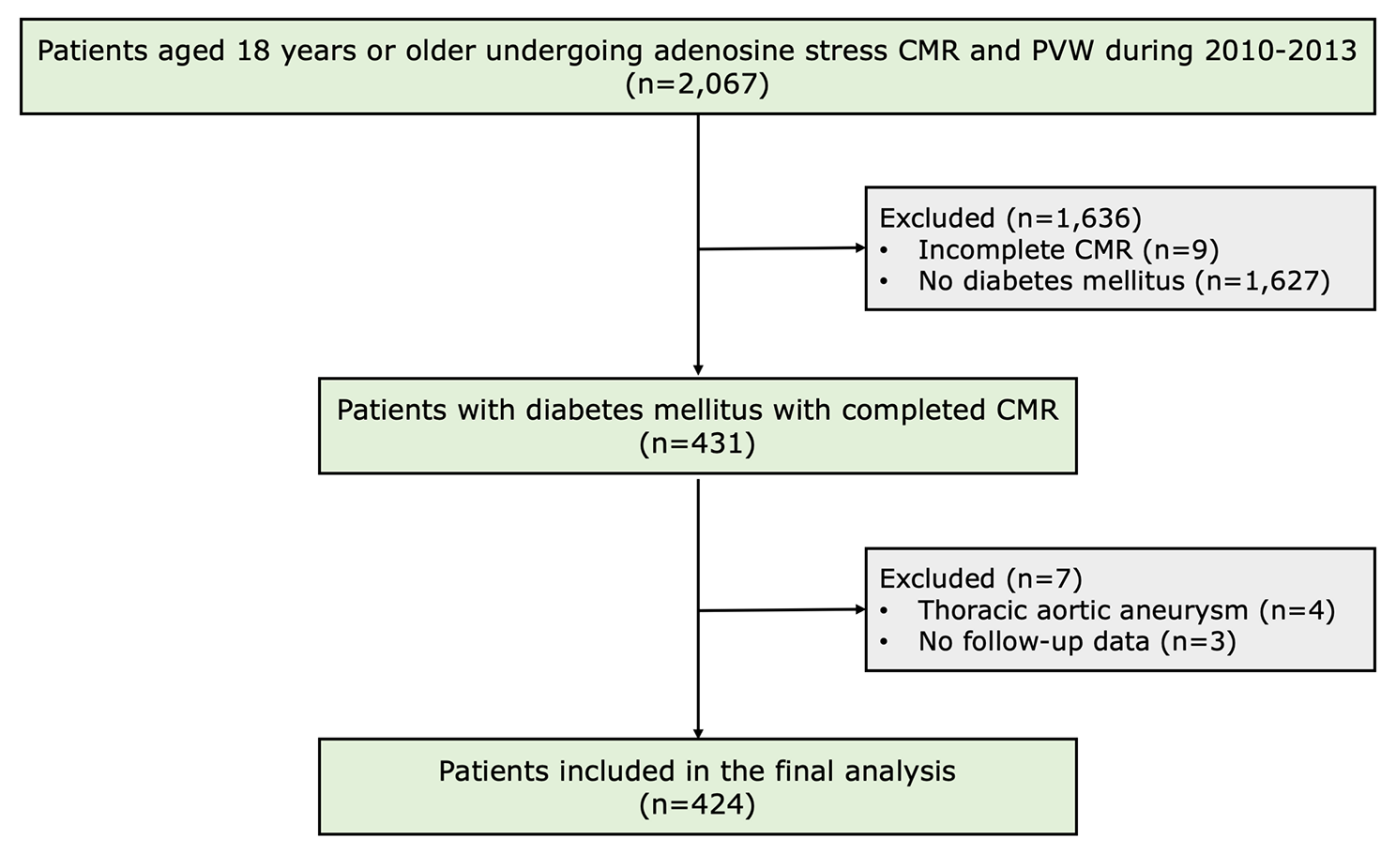
Study Flow Chart

A total of 431 patients with diabetes mellitus and suspected myocardial ischemia completed CMR protocol. Four patients were excluded due to having an aortic aneurysm, and three patients were excluded due to a loss of follow-up data. Ultimately, 424 patients were included in the final analysis (Fig. 1). No patient was excluded on the basis of CMR image quality. Baseline patient characteristics for all participants are summarized in Table 1, and a comparison is presented between those with elevated and non-elevated PWV. The mean age of patients was 70.5 years, and 45.5% were male. One hundred and seventeen patients had known CAD including 32 with a history of MI. Mean LVEF was 67.5%. Myocardial ischemia was present in 137 patients (32.2%) and LGE was present in 116 patients (27.3%). Mean PWV was 12.16 ± 6.28 m/s. Patients with elevated PWV (> 12.16 m/s) were older, had higher systolic blood pressure, and had a higher prevalence of CAD risk factors and microvascular complications than those with non-elevated PWV (≤ 12.16 m/s). There was no significant difference in LVEF, the prevalence of myocardial ischemia, or LGE between those with and without elevated PWV. Supplemental Table 1 shows univariable and multivariable binary logistic regression analyses for identifying predictors of elevated PWV. The multivariable analysis demonstrated that age, systolic blood pressure, hypertension, known CAD, and microvascular complications were independently associated with elevated PWV.

**Table 1:** Baseline Characteristics for All Patients, Compared Between the Elevated and Non-Elevated PWV Groups

	Total (n = 424)	Elevated PWV^a^ (n = 165)	Non-elevated PWV^b^ (n = 259)	p-value
Age (years) Male Body mass index (kg/m^2^) Systolic BP (mmHg) Diastolic BP (mmHg) Heart rate (beats/minute)	70.5 ± 9.8 193 (45.5) 27.4 ± 4.7 138.1 ± 19.6 70.3 ± 11.9 78.3 ± 14.0	73.7 ± 8.8 73 (44.2) 26.9 ± 4.3 142.9 ± 20.3 69.6 ± 12.3 79.0 ± 12.3	68.5 ± 10.0 120 (46.3) 27.7 ± 4.9 135.1 ± 18.6 70.8 ± 11.8 77.8 ± 13.4	***< 0.001*** 0.67 0.10 ***< 0.001*** 0.33 0.38
Clinical history				
Hypertension Hyperlipidemia Coronary artery disease Myocardial infarction Prior revascularization PCI CABG Ischemic stroke Cigarette smoker Chest pain Dyspnea Heart failure Microvascular complications Retinopathy Nephropathy Peripheral neuropathy	397 (93.4) 365 (85.9) 117 (27.5) 32 (7.5) 67 (15.8) 51 (12.0) 17 (4.0) 38 (8.9) 62 (14.6) 171 (40.3) 243 (57.3) 57 (13.4) 165 (38.9) 113 (26.7) 81 (19.1) 37 (8.7)	164 (99.4) 150 (90.9) 55 (33.3) 10 (6.1) 33 (20.0) 23 (13.9) 11 (6.7) 13 (7.9) 22 (13.3) 60 (36.4) 108 (65.5) 30 (18.2) 79 (47.9) 50 (30.3) 43 (26.1) 18 (10.9)	233 (90.0) 215 (83.0) 62 (23.9) 22 (8.5) 34 (13.1) 28 (10.8) 6 (2.3) 25 (9.7) 40 (15.4) 111 (42.9) 135 (52.1) 27 (10.4) 86 (33.2) 63 (24.3) 38 (14.7) 19 (7.3)	***< 0.001*** ***0.02*** ***0.03*** 0.36 0.06 0.34 ***0.03*** 0.53 0.55 0.18 ***0.01*** ***0.02*** ***0.003*** 0.17 ***0.004*** 0.20
Laboratory results				
Fasting plasma glucose (mg/dL) HbA1c (%)	140.5 ± 48.6 7.4 ± 1.4	141.7 ± 48.6 7.4 ± 1.1	139.8 ± 48.7 7.4 ± 1.6	0.69 0.72
Medications				
ACE inhibitor or ARB Antiplatelet Beta blocker Calcium channel blocker Statin Oral hypoglycemic drug Metformin Sulfonylurea Thiazolidinedione DPP 4 inhibitor Glinide GLP 1 receptor agonists Alpha-glucosidase inhibitor Average number of oral hypoglycemic drugs Insulin	238 (56.0) 285 (67.1) 258 (60.7) 164 (38.6) 292 (68.7) 322 (75.8) 244 (57.5) 170 (40.1) 42 (9.9) 67 (15.8) 2 (0.5) 1 (0.2) 14 (3.3) 1.27 ± 1.01 80 (18.8)	94 (57.0) 119 (72.1) 101 (61.2) 68 (41.2) 114 (69.1) 131 (79.4) 99 (60.0) 71 (43.0) 19 (11.5) 25 (15.2) 1 (0.6) 0 (0) 7 (4.0) 1.35 ± 1.02 33 (20.0)	144 (55.6) 166 (64.1) 157 (60.6) 96 (37.1) 178 (68.7) 191 (73.7) 145 (55.9) 99 (38.2) 23 (8.9) 42 (16.2) 1 (0.3) 1 (0.3) 7 (3.0) 1.23 ± 1.02 47 (18.1)	0.78 0.09 0.90 0.40 0.93 0.18 0.42 0.36 0.41 0.89 1.00 1.00 0.41 0.25 0.63
Electrocardiography				
Q wave	69 (16.3)	30 (18.2)	39 (15.1)	0.40
CMR				
LVEDV index (ml/m^2^)	76.3 ± 27.3	73.8 ± 24.5	77.9 ± 28.9	0.14
LVESV index (ml/m^2^)	28.4 ± 28.3	26.6 ± 25.6	29.6 ± 29.8	0.28
LV mass index (g/m^2^)	52.2 ± 16.5	52.5 ± 14.5	52.0 ± 17.6	0.74
LV ejection fraction (%)	67.5 ± 15.6	68.6 ± 16.0	66.8 ± 15.4	0.25
Myocardial ischemia present	137 (32.2)	59 (35.8)	78 (30.1)	0.22
Average number of ischemic segments^c^	6.6 ± 3.8	6.5 ± 3.6	6.7 ± 3.8	0.81
LGE present	116 (27.3)	45 (27.3)	71 (27.4)	0.98
Average number of LGE segments^d^	4.6 ± 2.5	4.5 ± 2.4	4.7 ± 2.5	0.41
PWV (m/s)	12.16 ± 6.28	17.44 ± 7.12	8.80 ± 1.81	***< 0.001***

Values are number (percentages) or mean ± SD. **Bold-italic** values are < 0.05

^a^Defined as PWV > 12.16 m per second

^b^Defined as PWV ≤ 12.16 m per second

^c^Only in patients with myocardial ischemia

^d^Only in patients with LGE

**Abbreviations:** ACE, angiotensin-converting enzyme; ARB, angiotensin II receptor blocker; BP, blood pressure; CABG, coronary artery bypass graft; CMR, cardiac magnetic resonance; DPP, dipeptidyl peptidase; ECG, electrocardiography; GLP, glucagon like peptide; LGE, late gadolinium enhancement; LV, left ventricular; LVEDV, left ventricular end-diastolic volume; LVESV, left ventricular end-systolic volume; MACE, major adverse cardiovascular events; m/s, metre per second; PCI, percutaneous coronary intervention; PWV, pulse wave velocity

### Patient outcomes

**Fig. 2:**
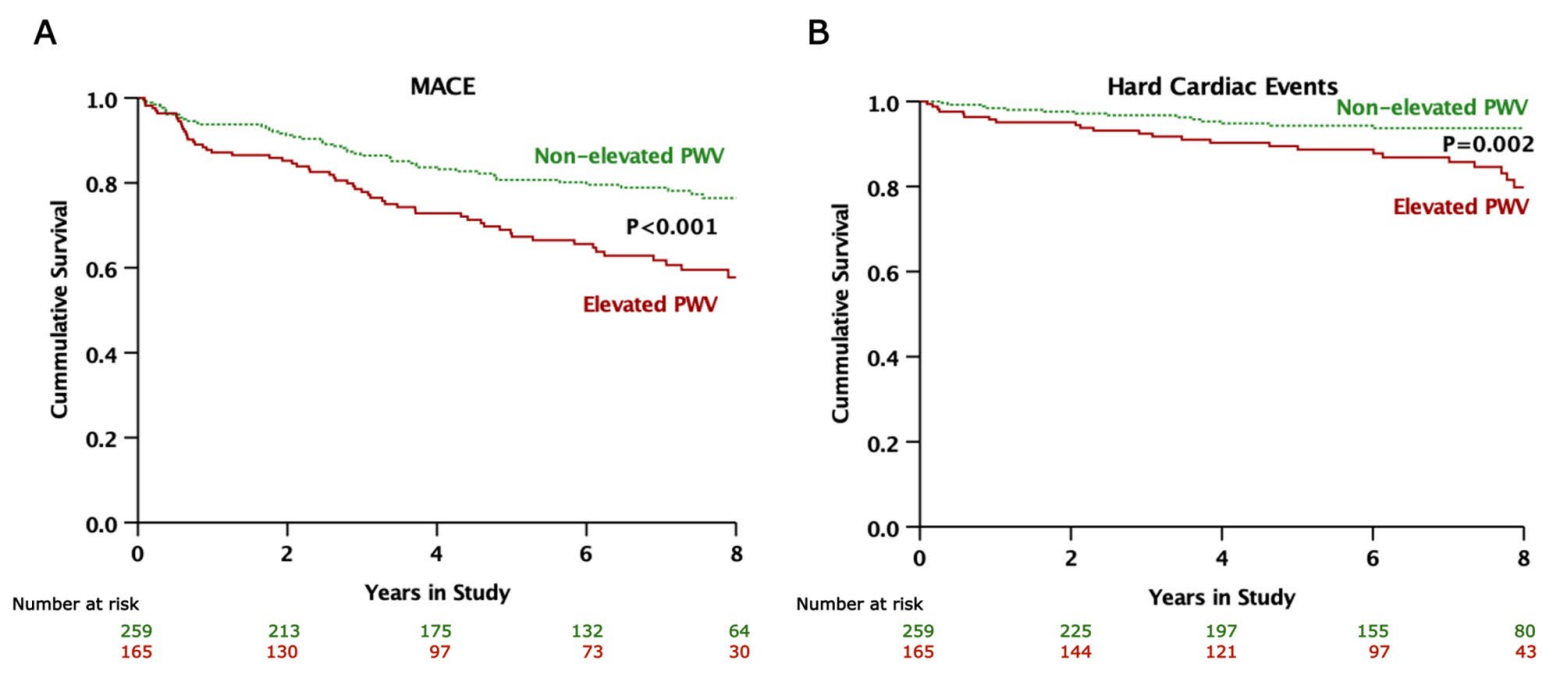
Kaplan-Meier survival analysis showing the unadjusted cumulative incidence of MACE (**A**) and hard cardiac events (**B**) compared between those with elevated and non-elevated PWV. Patients with elevated PWV had significantly higher rates of MACE and hard cardiac events compared to patients with non-elevated PWV **Abbreviations:** MACE, major adverse cardiovascular events; PWV, pulse wave velocity

During a median follow-up period of 7.2 years (interquartile range [IQR] 4.6, 8.9 years), MACE occurred in 114 patients (26.8%), including 40 hard cardiac events (9.4%). Clinical events in the study cohort are detailed in Table 2. Patients with elevated PWV exhibited significantly higher rates of MACE and hard cardiac events, with unadjusted hazard ratios (HR) of 2.14 (95%CI 1.48, 3.09), p < 0.001 and 2.69 (95%CI 1.42, 5.10), p = 0.002, respectively. After adjusting for age, gender, baseline CAD status, and HbA_1C_ level – factors previously associated with cardiac events in prior publications [4, 24, 25] – the significant differences between groups persisted for both MACE (p = 0.001) and hard cardiac events (p = 0.008). Kaplan-Meier survival analysis showed that patients with elevated PWV had a significantly higher incidence of both MACE (Fig. 2A) and hard cardiac events (Fig. 2B) compared to patients with non-elevated PWV. When patients were divided into tertiles based on PWV (Supplemental Fig. 2), those in the 3rd tertile exhibited significantly higher rates of both MACE and hard cardiac events compared to those in the 1st and 2nd tertiles. Specifically, for MACE, the HR were as follows: 3rd tertile versus 1st tertile: HR 2.12 (95%CI 1.37, 3.27), p = 0.001; 3rd tertile versus 2nd tertile: HR 2.65 (95%CI 1.65, 4.27), p < 0.001. Similarly, for hard cardiac events, the HR were: 3rd tertile versus 1st tertile: HR 2.17 (95%CI 1.06, 4.46), p = 0.03; 3rd tertile versus 2nd tertile: HR 3.73 (95%CI 1.52, 9.17), p = 0.004). Notably, patients in the 1st and 2nd tertiles showed no significant differences in the rates of MACE (with the 1st tertile as the reference: HR 0.78 [95%CI 0.46, 1.34], p = 0.37) and hard cardiac events (with the 1st tertile as the reference: HR 0.57 [95%CI 0.21, 1.55], p = 0.27).

**Table 2:** Cardiovascular Events in All Patients, Compared Between the Elevated and Non-Elevated PWV Groups

	Total (n = 424) n (%)	Elevated PWV (n = 165) n (%)	Non-elevated PWV (n = 259) n (%)	Unadjusted Model		Adjusted Model^a^	
				HR (95% CI)	p-value	HR (95% CI)	p-value
Cardiac mortality	20 (4.7)	14 (8.5)	6 (2.3)	3.75 (1.44, 9.75)	***0.01***	2.97 (1.09, 8.07)	***0.03***
Nonfatal myocardial infarction	26 (6.1)	14 (8.5)	12 (4.6)	1.87 (0.86, 4.04)	0.11	1.75 (0.77, 3.97)	0.18
Hospitalization for heart failure	54 (12.7)	32 (19.4)	22 (13.8)	2.39 (1.39, 4.12)	***0.002***	1.87 (1.05, 3.33)	***0.03***
Coronary revascularization	46 (10.8)	25 (15.2)	21 (8.1)	1.93 (1.08, 3.45)	***0.03***	1.61 (0.85, 3.02)	0.14
Ischemic stroke	27 (6.4)	17 (10.3)	10 (3.9)	2.81 (1.29, 6.14)	***0.01***	2.76 (1.21, 6.28)	***0.01***
MACE^b^	114^d^ (26.8)	63 (38.2)	51 (19.7)	2.14 (1.48, 3.09)	***< 0.001***	1.92 (1.29, 2.86)	***0.001***
Hard cardiac events^c^	40^e^ (9.4)	25 (15.2)	15 (5.8)	2.69 (1.42, 5.10)	***0.002***	2.50 (1.27, 4.92)	***0.008***

**Bold-italic** values are < 0.05

^a^Adjusted for age, gender, baseline coronary artery disease status, and HbA1c level

^b^MACE was defined as the composite of cardiac mortality, nonfatal myocardial infarction, hospitalized for heart failure, coronary revascularization, and ischemic stroke

^c^Hard cardiac events were defined as the composite of cardiac mortality and nonfatal myocardial infarction

^d^Forty-two patients experienced more than one cardiovascular event

^e^Six patients experienced more than one cardiovascular event

**Abbreviations:** CI, confidence interval; HR, hazard ratio; MACE, major adverse cardiovascular events; PWV, pulse wave velocity

### Predictors of MACE and hard cardiac events

The results of multivariable Cox survival analyses for MACE (Table 3) and hard cardiac events (Table 4), analyzing PWV both as a categorized variable (> 12.16 m/s, model 1) and as a continuous variable (model 2), demonstrated that PWV was an independent predictor for MACE (p = 0.01 for model 1, p = 0.003 for model 2) and hard cardiac events (p = 0.01). Other independent predictors of MACE included prior revascularization (p < 0.001), history of heart failure (p < 0.001), ischemic burden (per segment) (p < 0.001), and the presence of LGE (p = 0.02 for model 1 and p = 0.01 for model 2). Additional independent predictors for hard cardiac outcomes were prior revascularization (p = 0.02), presence of microvascular complications (p = 0.03 for model 1 and p = 0.02 for model 2), LV mass index (p = 0.02 for model 1), and presence of LGE (p = 0.02 for model 1 and p = 0.03 for model 2).

**Table 3:** Univariable and Multivariable Cox Regression Analyses for Identifying Independent Predictors of MACE.

	Univariable Analysis		Multivariable Analysis			
			Model 1^a^		Model 2^b^	
	HR (95% CI)	p-value	HR (95% CI)	p-value	HR (95% CI)	p-value
Age (years) Male Body mass index (kg/m^2^) Systolic BP (mmHg) Diastolic BP (mmHg) Heart rate (beats/minute) Hypertension Hyperlipidemia Coronary artery disease Myocardial infarction	1.004 (0.99, 1.02) 1.40 (0.97, 2.02) 0.95 (0.91, 0.99) 0.99 (0.99, 1.01) 0.99 (0.97, 1.00) 1.00 (0.98, 1.01) 3.40 (0.84, 13.77) 1.43 (0.77, 2.67) 3.05 (2.11, 4.41) 1.88 (1.05, 3.33)	0.71 0.07 ***0.01*** 0.46 ***0.04*** 0.69 0.86 0.26 ***< 0.001*** ***0.03***				
Prior revascularization	3.65 (2.46, 5.42)	***< 0.001***	2.88 (1.86, 4.47)	***< 0.001***	2.72 (1.74, 4.25)	***< 0.001***
Ischemic stroke Cigarette smoker Chest pain Dyspnea	1.23 (0.66, 2.29) 1.22 (0.75, 2.00) 1.03 (0.71, 1.49) 1.26 (0.86, 1.84)	0.52 0.42 0.89 0.24				
Heart failure	3.81 (2.53,5.75)	***< 0.001***	2.69 (1.73, 4.17)	***< 0.001***	2.87 (1.85, 4.44)	***< 0.001***
Microvascular complications Fasting plasma glucose HbA1c Q wave on ECG LVEDV index LVESV index LV mass index LV ejection fraction (%) Myocardial ischemia present	1.42 (0.98, 2.05) 1.003 (1.00, 1.01) 1.06 (0.95, 1.19) 1.96 (1.27, 3.02) 1.01 (1.01, 1.02) 1.01(1.01, 1.02) 1.03 (1.02, 1.03) 0.98 (0.97, 0.99) 2.85 (1.97, 4.13)	0.06 0.06 0.29 ***0.002*** ***< 0.001*** ***< 0.001*** ***< 0.001*** ***< 0.001*** ***< 0.001***				
Ischemic burden (per segment) LGE present	1.11 (1.07, 1.15) 3.04 (2.10, 4.42)	***< 0.001*** ***< 0.001***	1.08 (1.04, 1.13) 1.62 (1.05, 2.48)	***< 0.001*** ***0.02***	1.08 (1.04, 1.13) 1.70 (1.10, 2.61)	***< 0.001*** ***0.01***
LGE burden (per segment)	1.14 (1.08, 1.20)	***< 0.001***				
Elevated PWV (> 12.16 m/s)	2.14 (1.48, 3.09)	***< 0.001***	1.70 (1.16, 2.48)	***0.01***		
PWV (m/s)	1.04 (1.02, 1.06)	***< 0.001***			1.03 (1.01, 1.05)	***0.003***

**Bold-italic** values are < 0.05

^a^PWV was included as a categorical variable (elevated or non-elevated)

^b^PWV was included as a continuous variable (m/s)

**Abbreviations:** CI, confidence interval; BP, blood pressure; ECG, electrocardiography; HR, hazard ratio; LGE, late gadolinium enhancement; LV, left ventricular; LVEDV, left ventricular end-diastolic volume; LVESV, left ventricular end-systolic volume; MACE, major adverse cardiovascular events; PWV, pulse wave velocity

**Table 4:** Univariable and Multivariable Cox Regression Analyses for Identifying Independent Predictors of Hard Cardiac Events

	Univariable Analysis		Multivariable Analysis			
			Model 1^a^		Model 2^b^	
	HR (95% CI)	p-value	HR (95% CI)	p-value	HR (95% CI)	p-value
Age (years) Male Body mass index (kg/m^2^) Systolic BP (mmHg) Diastolic BP (mmHg) Heart rate (beats/minute) Hypertension Hyperlipidemia Coronary artery disease Myocardial infarction	1.01 (0.98, 1.04) 1.31 (0.71, 2.44) 0.91 (0.85, 0.99) 0.99 (0.97, 1.01) 0.98 (0.95, 1.00) 1.00 (0.98, 1.02) 2.12 (0.29, 15.44) 1.32 (0.47, 3.70) 2.86 (1.54, 5.32) 1.89 (0.74, 4.82)	0.69 0.39 0.18 0.22 0.05 0.94 0.46 0.60 ***0.001*** 0.18				
Prior revascularization	3.76 (1.98, 7.14)	***< 0.001***	2.28 (1.12, 4.62)	***0.02***	2.30 (1.14, 4.66)	***0.02***
Ischemic stroke Cigarette smoker Chest pain Dyspnea Heart failure	0.57 (0.14, 2.36) 1.04 (0.43, 2.47) 1.15 (0.62, 2.14) 1.02 (0.55, 1.91) 3.07 (1.53, 6.14)	0.44 0.94 0.66 0.95 ***0.002***				
Microvascular complications	2.46. (1.30, 4.68)	***0.006***	2.07 (1.08, 3.96)	***0.03***	2.09 (1.09, 4.01)	***0.02***
Fasting plasma glucose HbA1c Q wave on ECG LVEDV index LVESV index	1.004 (0.99, 1.01) 1.13 (0.96, 1.34) 1.94 (0.95, 3.96) 1.01 (1.004, 1.02) 1.01 (1.004, 1.02)	0.12 0.15 0.71 ***0.01*** ***0.002***				
LV mass index	1.03 (1.02, 1.05)	***< 0.001***	1.02 (1.003, 1.04)	***0.02***		
LV ejection fraction (%) Myocardial ischemia present Ischemic burden (per segment)	0.98 (0.96, 0.99) 2.41 (1.30, 4.48) 1.10 (1.04, 1.18)	***0.004*** ***0.01*** ***0.001***				
LGE present	4.11 (2.20, 7.68)	***< 0.001***	2.31 (1.11, 4.82)	***0.02***	2.24 (1.07, 4.72)	***0.03***
LGE burden (per segment)	1.16 (1.07, 1.26)	***< 0.001***				
Elevated PWV (> 12.16 m/s)	2.69 (1.42, 5.10)	***0.002***	2.22 (1.16, 4.26)	***0.01***		
PWV (m/s)	1.05 (1.02, 1.08)	***0.001***			1.04 (1.01, 1.08)	***0.01***

**Bold-italic** values are < 0.05

^a^PWV was included as a categorical variable (elevated or non-elevated). (> 12.16 m/s)

^b^PWV was included as a continuous variable (m/s)

**Abbreviations:** CI, confidence interval; BP, blood pressure; ECG, electrocardiography; HR, hazard ratio; LGE, late gadolinium enhancement; LV, left ventricular; LVEDV, left ventricular end-diastolic volume; LVESV, left ventricular end-systolic volume; MACE, major adverse cardiovascular events; PWV, pulse wave velocity

### Incremental prognostic value of PWV

The incremental prognostic value resulting from the inclusion of PWV in the predictive algorithm for MACE and hard cardiac events is presented in Table 5. When evaluating prognosis hierarchically (clinical only; clinical + other CMR parameters; and clinical + other CMR parameters + PWV), PWV demonstrated increased incremental prognostic value for both MACE (incremental χ²: 7.54, p = 0.006) and hard cardiac events (incremental χ²: 5.99, p = 0.01).

**Table 5:** Incremental Prognostic Value ﻿of PWV for Predicting MACE and Hard Cardiac Events

MACE	Global 𝛘^2^	𝛘^2^ of difference	p-value of difference with immediately above model
Clinical model 1^a^	94.45	N/A	N/A
Clinical model 1^a^ + Ischemic burden	117.68	16.13	***< 0.001***
Clinical model 1^a^ + Ischemic burden + LGE present	122.57	3.82	0.05
Clinical model 1^a^ + Ischemic burden + LGE present + Elevated PWV	129.15	7.54	***0.006***
**Hard Cardiac Events**	**Global 𝛘** ^**2**^	**𝛘**^**2**^ **of difference**	**p-value of difference with immediately above model**
Clinical model 2^b^	27.50	N/A	N/A
Clinical model 2^b^ + LV mass index	40.79	9.64	***0.002***
Clinical model 2^b^ + LV mass index + LGE present	45.65	4.45	***0.03***
Clinical model 2^b^ + LV mass index + LGE present + Elevated PWV	50.75	5.99	***0.01***

**Bold-italic** values are < 0.05

^a^Clinical model 1: Stepwise selection was used to determine significant variables showing an association with MACE based on multivariable analysis (p < 0.05). These variables included prior revascularization and a history of heart failure

^b^Clinical model 2: Stepwise selection was used to determine significant variables showing an association with hard cardiac events based on multivariable analysis (p < 0.05). These variables included prior revascularization and microvascular complications

**Abbreviations:** LGE, late gadolinium enhancement; LV, left ventricular; MACE, major adverse cardiovascular events; N/A, not available; PWV, pulse wave velocity; 𝛘2, chi-square

### PWV and myocardial ischemia

**Fig. 3:**
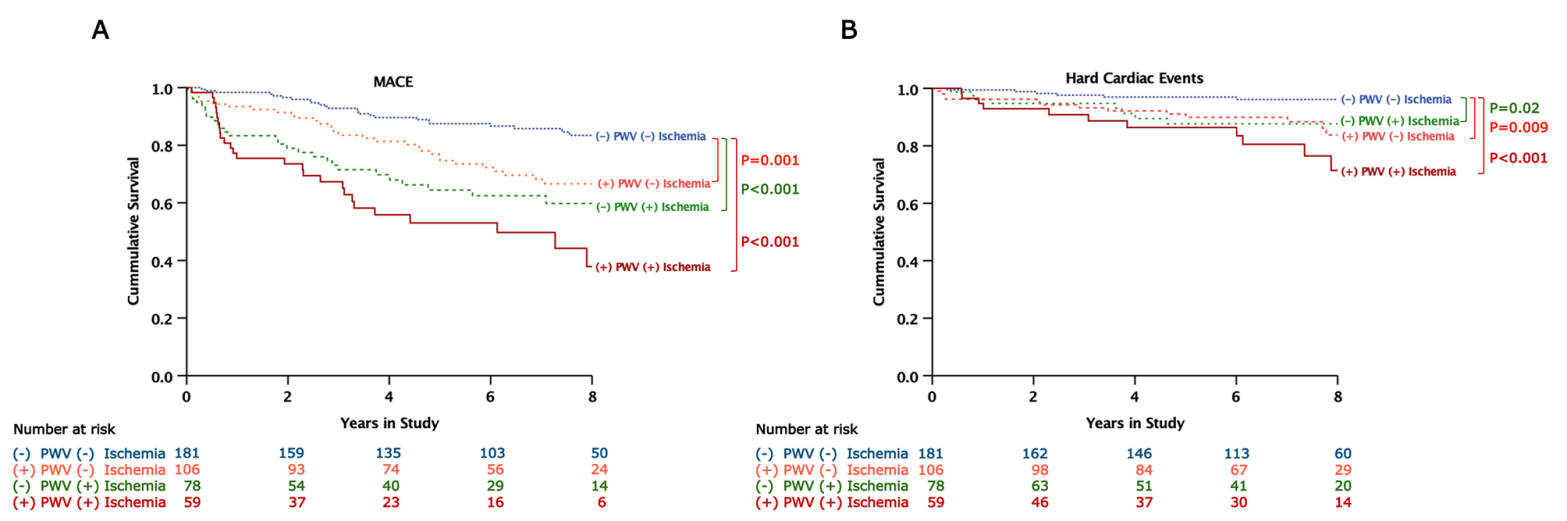
Kaplan-Meier survival analysis showing the unadjusted cumulative incidence of MACE (**A**) and hard cardiac events (**B**) compared among various combinations of PWV and myocardial ischemia status. Patients with elevated PWV only (orange) or positive myocardial ischemia only (green) had significantly higher rates of MACE and hard cardiac events compared to patients with non-elevated PWV and no ischemia (blue). Patients with coexisting elevated PWV and myocardial ischemia (red) had the highest rates of MACE and hard cardiac events **Abbreviations:** MACE, major adverse cardiovascular events; PWV, pulse wave velocity

For this analysis, we divided patients into 4 groups, as follows: group 1 – non-elevated PWV and no myocardial ischemia; group 2 – elevated PWV and no myocardial ischemia; group 3 – non-elevated PWV and presence of myocardial ischemia; and group 4 – elevated PWV and presence of myocardial ischemia. The results of our analysis revealed that compared to patients with non-elevated PWV and no myocardial ischemia (group 1), patients with elevated PWV only or the presence of myocardial ischemia only (group 2 or 3) exhibited significantly higher rates of MACE (group 2: HR 2.46 [95% CI 1.45, 4.16], p = 0.001; group 3: HR 3.32 [95% CI 1.91, 5.76], p < 0.001) and hard cardiac events (group 2: HR 3.19 [95% CI 1.28, 8.01], p = 0.009; group 3: HR 3.00 [95% CI 1.09, 8.30], p = 0.02). Patients with elevated PWV and presence of myocardial ischemia (group 4) had over six times the rates of MACE (HR 6.16 [95%CI 3.58, 10.61], p < 0.001) and hard cardiac events (HR 6.29 [95%CI 2.47, 15.99], p < 0.001) compared to those with non-elevated PWV and no myocardial ischemia (group 1) (Fig. 3).

### Subgroup analyses for the primary outcome

**Fig. 4:**
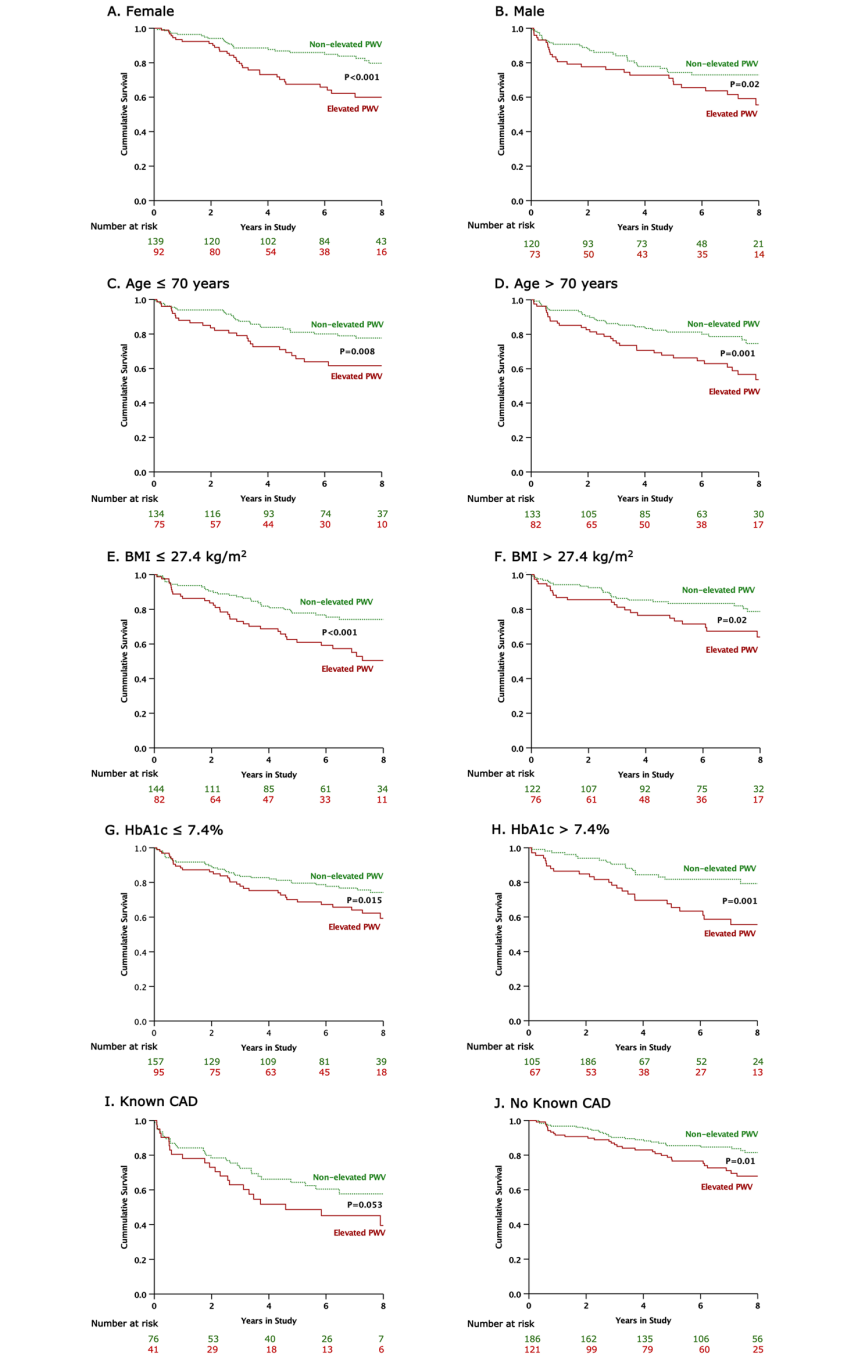
Kaplan-Meier subgroup analyses of the primary outcome, including comparisons between genders (**A**, **B**); age levels (**C**, **D**); BMI levels (**E**, **F**); HbA1c levels (**G**, **H**); and, known CAD and no known CAD (**I**, **J**) **Abbreviations:** BMI, body mass index; CAD, coronary artery disease; HbA1c, glycated hemoglobin; PWV, pulse wave velocity

Figure 4 depicts Kaplan-Meier survival curves for patients with and without elevated PWV, compared between females and males (Fig. 4A, 4B); between patients aged ≤70 and >70 years (Fig. 4C, 4D); between patients with a BMI of ≤27.4 kg/m2 and >27.4 kg/m2 (Fig. 4E, 4F); between patients with an HbA1c level of ≤7.4% and >7.4% (Fig. 4G, 4H); and between patients with and without known CAD (Fig. 4I, 4J). In all 10 patient subgroups, elevated PWV (greater than the mean PWV in each subgroup) was significantly associated with MACE. Among patients with known CAD, the difference in MACE rates between patients with elevated PWV and those with non-elevated PWV showed a trend toward significance, but it did not reach statistical significance (p = 0.053).

## Discussion

This study showed that increased aortic stiffness, measured by CMR-based aortic PWV, predicted MACE and hard cardiac events in patients with diabetes mellitus who underwent adenosine stress perfusion CMR over and beyond traditional cardiovascular risk factors, myocardial ischemia, and LGE. Furthermore, adding aortic stiffness provided incremental prognostic value with clinical data and CMR parameters. Taken together, the results of this study suggest that aortic stiffness measurement could be incorporated into a stress perfusion CMR protocol to improve risk prediction in this group.

Diabetes mellitus affects approximately 10% of adults worldwide [26], is a significant risk factor for CAD, and is associated with all-cause and cardiovascular mortality [27]. Previous studies established the prognostic value of aortic stiffness in general population [3], and in patients with CAD [10, 28], arterial hypertension [29], end-stage renal disease [30], and diabetes mellitus [4, 25]. Patients with diabetes mellitus had a greater risk of elevated PWV than individuals without diabetes mellitus [31, 32]. Cardoso, et al. reported that aortic stiffness as measured by carotid-femoral PWV yielded cardiovascular risk prediction independent of standard risk factors and ambulatory blood pressure [4]. In our study, the mean PWV among overall patients with diabetes mellitus was 12.16 m/s, which is higher than the mean PWV reported in general population [33]. Patients with elevated PWV were significantly older and had a significantly higher prevalence of arterial hypertension, hyperlipidemia, microvascular complications, and systolic blood pressure than those with non-elevated PWV, which is consistent with previous studies [3–5]. Aortic stiffness is an integrated indicator of the cumulative damage of aging and cardiovascular risk factors on the arterial wall over time [34]. In patients with diabetes mellitus, aortic stiffness appears to be accelerated by long-term hyperglycemia and the formation of advanced glycation end products on the arterial wall, which results in endothelial dysfunction that causes diastolic capillary dysfunction, shrinkage or sparse distribution, and stiffening of the artery wall [25]. Additionally, a recent study by Zheng, et al. found that an increase in arterial stiffness appeared to precede an increase in fasting blood glucose, and arterial stiffness was associated with a higher risk of incident diabetes mellitus independent of traditional risk factors [35]. Overall, aortic stiffness is a very important cardiovascular risk marker, as well as a crucial prognostic predictor.

CMR-based PWV was reported to be superior to carotid-femoral PWV relative to the accuracy of measurement of the aortic length, which was validated by invasive pressure recordings [7]. CMR-based PWV measurement was also reported to have excellent reproducibility [7, 17]. PWV is measured during the waiting period between the stress perfusion and LGE studies, which is an added benefit of including it in the stress perfusion CMR protocol because the inclusion of PWV does not increase the total scan time. The prognostic value of CMR-based PWV in patients who underwent adenosine stress CMR has been reported [10]. The prognostic value of CMR-based PWV in asymptomatic patients with diabetes mellitus has also been reported [11]. However, the prognostic value of CMR-based PWV in patients with diabetes mellitus who underwent adenosine stress CMR has not been studied. In our study, patients with elevated PWV demonstrated significantly higher rates of MACE and hard cardiac events compared to those with non-elevated PWV. This significant finding remained consistent in multiple subanalysis comparisons between groups for age, gender, BMI, and HbA1c level. In the subgroup analysis of patients with known CAD, individuals with elevated PWV showed a strong trend toward a significantly higher rate of MACE compared to those with non-elevated PWV; however, the difference between groups was just short of achieving statistical significance (p = 0.053). This may be explained by the relatively small number of patients in the CAD subgroup (n = 117), or there may be other confounding factors specific to this subgroup. Further study in this area of interest is warranted. Our results showed PWV to be a strong independent predictor of MACE and hard cardiac events that exceeds the predictive power of traditional cardiovascular risk factors, ECG, and other CMR parameters. PWV was also shown to confer significantly increased incremental prognostic value for predicting cardiovascular events. This was a promising role of PWV to add a prognostic value over LVEF, myocardial ischemia, and LGE, which were established predictors for future cardiovascular events including mortality [2, 18].

Arterial stiffness is an important risk factor and a useful prognostic marker for cardiovascular events, including CAD. Arterial stiffness contributes to myocardial ischemia through the loss of coronary artery compliance rather than a change in the reflecting time (an early arrival of wave reflections in systole instead of diastole) [36]. Our results revealed aortic stiffness and myocardial ischemia to be independent predictors of MACE. Moreover, patients with diabetes mellitus with coexisting elevated PWV and myocardial ischemia also experienced the worst clinical outcome. Thus, we believe that assessing aortic stiffness and myocardial ischemia in a single CMR study is an enhanced strategy for evaluating patients with diabetes mellitus with known or suspected CAD.

This study has some mentionable limitations. First, the retrospective design of this study rendered it vulnerable to missing or incomplete data and to certain biases. For example, we were not able to include duration of diabetes mellitus in our analysis since this data was not consistently available. Secondly, we divided the group of patients using the mean PWV instead of the more common approach of utilizing the median. However, our data revealed that the mean and median PWV values were relatively close (mean ± SD: 12.16 ± 6.28 m/s, median [IQR]: 10.58 [8.41, 13.96] m/s), and the main findings appear to remain unchanged. Third, although our multivariable regression models were adjusted for multiple covariates, there may be other confounding factors that influence the relationship between PWV and the incidence of cardiovascular events. Fourth, this study included Asian patients with a mean age of 70 years, so the generalizability of our findings to younger patients and to patients of other races should be performed with caution. Fourth and finally, the fact that we used the mean PWV as the cut-off value may make translating our findings into clinical practice challenging. However, since there is no optimal cut-off value for PWV in patients with diabetes mellitus, further study is warranted to identify a cut-off or cut-offs appropriate to this specific clinical setting.

In conclusion, aortic stiffness by CMR independently predicts MACE and hard cardiac events and confers significant incremental prognostic value in patients with diabetes mellitus with suspected myocardial ischemia. Aortic stiffness measurement could potentially be considered as part of a stress perfusion CMR protocol to enhance risk prediction in patients with diabetes mellitus.

### Electronic supplementary material

Below is the link to the electronic supplementary material.

Supplementary Material 1

## Data Availability

The datasets used and/or analyzed during the current study are available from the corresponding author on reasonable request.

## Abbreviations

CAD: Coronary artery disease
CMR: Cardiovascular magnetic resonance
EF: Ejection fraction
FOV: Field of view
LGE: Late gadolinium enhancement
LV: Left ventricular/ventricle
MACE: Major adverse cardiovascular events
MI: Myocardial infarction
PWV: Pulse wave velocity
SSFP: Steady-state free precession
TE: Echo time
TR: Repetition time
VE-CMR: Velocity-encoded cardiovascular magnetic resonance
