# Supplementary material for: Aortic stiffness effectively risk stratifies diabetic patients with suspected myocardial ischemia undergoing vasodilatory stress perfusion cardiac magnetic resonance
Source: BMC Cardiovasc Disord. 2023 Oct 10;23:502. doi: 10.1186/s12872-023-03532-0 (PMC10566029; doi:10.1186/s12872-023-03532-0)
Supplement: Supplementary file 1 — Supplementary Material 1 [file 12872_2023_3532_MOESM1_ESM.docx]

**Supplemental Materials**

**Supplemental Table 1.** Univariable and Multivariable Binary Logistic Regression Analyses for Identifying Independent Predictors of Elevated PWV.

|  | Univariable Analysis | | Multivariable Analysis | |
| --- | --- | --- | --- | --- |
|  | HR (95% CI) | p-value | HR (95% CI) | p-value |
| Age (years)  Male  Body mass index (kg/m^2^)  Systolic BP (mmHg)  Diastolic BP (mmHg)  Heart rate (beats/minute)  Hypertension  Hyperlipidemia  Coronary artery disease  Myocardial infarction  Prior revascularization  Ischemic stroke  Cigarette smoker  Chest pain  Dyspnea  Heart failure  Microvascular complications  Fasting plasma glucose  HbA1c  ACE inhibitor or ARB  Antiplatelet  Beta blocker  Calcium channel blocker  Statin  Any oral hypoglycemic drug  Metformin  Sulfonylurea  Thiazolidinedione  DPP 4 inhibitor  Glinide  Alpha-glucosidase inhibitor  Number of oral hypoglycemic drugs  Insulin  Q wave on ECG  LVEDV index  LVESV index  LV mass index  LV ejection fraction (%)  Myocardial ischemia present  Ischemic burden (per segment)  LGE present  LGE burden (per segment) | 1.06 (1.04, 1.08)  0.92 (0.62, 1.36)  0.97 (0.93, 1.01)  1.02 (1.01, 1.03)  0.99 (0.98, 1.00)  1.01 (0.99, 1.02)  18.30 (2.46, 136.21)  2.05 (1.10, 3.81)  1.59 (1.03, 2.45)  0.70 (0.32, 1.51)  1.65 (0.98, 2.79)  0.80 (0.40, 1.61)  0.84 (0.48, 1.48)  0.76 (0.51, 1.14)  1.74 (1.16, 2.60)  1.91 (1.09, 3.35)  1.85 (1.24, 2.76)  1.001 (0.99, 1.005)  0.98 (0.85, 1.12)  1.06 (0.71, 1.57)  1.45 (0.95, 2.22)  1.03 (0.69, 1.53)  1.19 (0.80, 1.78)  1.02 (0.67, 1.55)  1.37 (0.86, 2.19)  1.18 (0.79, 1.75)  1.22 (0.82, 1.82)  1.34 (0.70, 2.54)  0.92 (0.54, 1.58)  1.57 (0.10, 25.33)  1.60 (0.55, 4.63)  1.12 (0.93, 1.36)  1.13 (0.69, 1.85)  1.25 (0.74, 2.11)  0.99 (0.98, 1.002)  0.99 (0.98, 1.003)  1.002 (0.99, 1.01)  1.008 (0.99, 1.02)  1.29 (0.85, 1.96)  1.02 (0.97, 1.07)  0.99 (0.64, 1.54)  0.99 (0.92, 1.07) | ***<0.001***  0.67  0.10  ***<0.001***  0.33  0.38  ***0.005***  ***0.02***  ***0.03***  0.36  0.06  0.53  0.55  0.18  ***0.01***  ***0.02***  ***0.003***  0.69  0.74  0.78  0.09  0.90  0.39  0.94  0.19  0.42  0.33  0.38  0.77  0.75  0.39  0.25  0.64  0.40  0.14  0.28  0.75  0.25  0.23  0.41  0.98  0.81 | 1.06 (1.04, 1.09)  1.02 (1.01, 1.03)  11.39 (1.48, 87.41)  1.89 (1.17, 3.04)  1.97 (1.27, 3.04) | ***<0.001***  ***0.006***  ***0.01***  ***0.01***  ***0.002*** |

**Bold-italic** values are <0.05.

**Abbreviations:** ACE, angiotensin-converting enzyme; ARB, angiotensin II receptor blocker; BP, blood pressure; **CI, confidence interval**; HR, hazard ratio; DPP, dipeptidyl peptidase-4; LGE, late gadolinium enhancement; LV, left ventricular; LVEDV, left ventricular end-diastolic volume; LVESV, left ventricular end-systolic volume; m/s, metre per second; PWV, pulse wave velocity.

**Supplemental Figure 1.** Measurement of PWV.

**
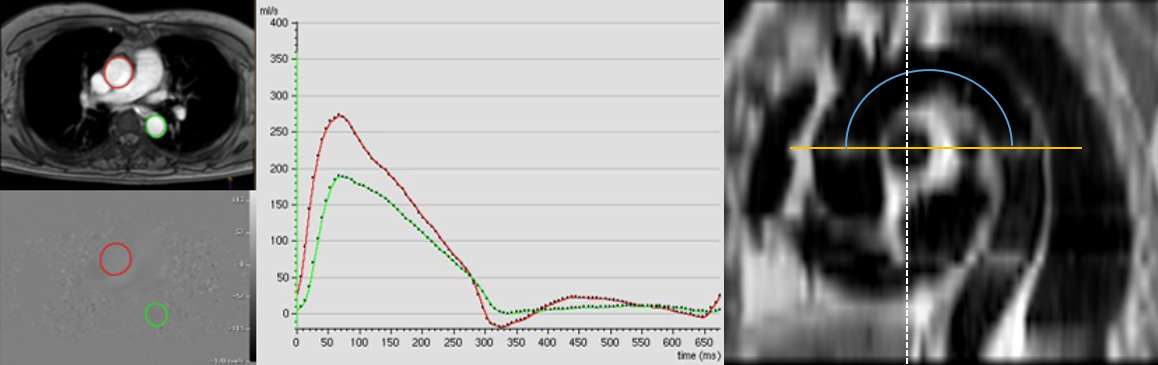
**

**Figure Legend:** Measurement of Time Delay between Pulse Waves and Aortic Path Length. Left: Through-plane VE-CMR at the mid-ascending (red circles) and mid-descending thoracic aorta (green circles). Middle: Corresponding flow measurement at the mid-ascending (red line) and mid-descending thoracic aorta (green line). Right: The measurement of aortic path length using a multiplanar reconstructed oblique sagittal view. Reprinted from IJC Heart & Vasculature, Vol 30, Kaolawanich Y, Boonyasirinant T, Impact of aortic stiffness by velocity-encoded magnetic resonance imaging on late gadolinium enhancement to predict cardiovascular events, Copyright (2020), with permission from Elsevier.

**Abbreviations:** VE-CMR, velocity-encoded cardiovascular magnetic resonance; PWV, pulse wave velocity.

**Supplemental Figure 2.** MACE and Hard Cardiac Events Divided by Tertiles of PWV.


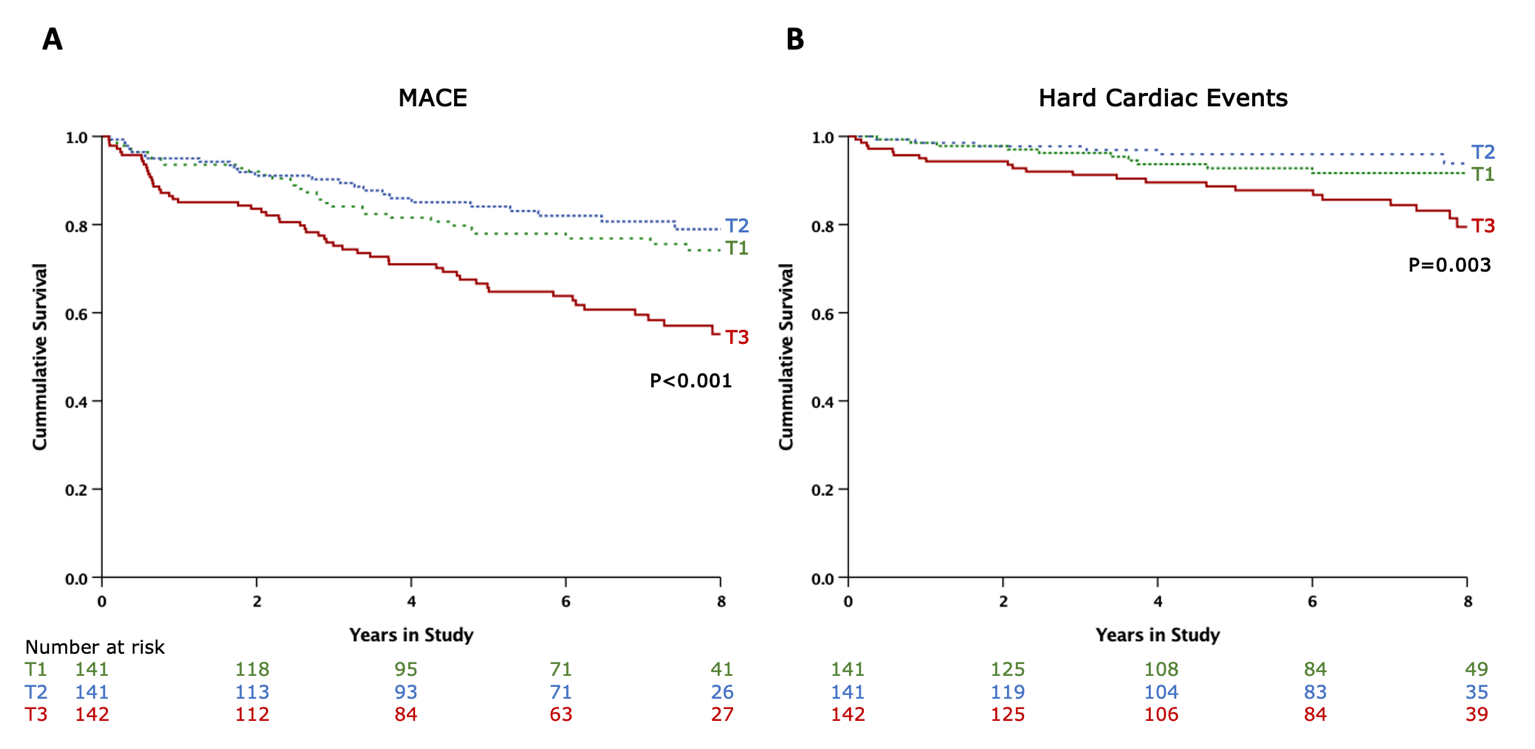


**Figure Legend:** Kaplan-Meier survival analysis depicting the unadjusted cumulative incidence of MACE **(A)** and hard cardiac events **(B)** compared between those within tertile PWV groups. Patients in the 3^rd^ tertile (T3: red) exhibited significantly higher rates of MACE and hard cardiac events when compared to patients in the 1^st^ (T1: green) and 2^nd^ tertiles (T2: blue).

**Abbreviations:** MACE, major adverse cardiovascular events; PWV, pulse wave velocity.
